# 2-(Benz­yloxy)iso­quinoline-1,3(2*H*,4*H*)-dione

**DOI:** 10.1107/S1600536813019934

**Published:** 2013-07-24

**Authors:** Yoshinobu Ishikawa, Soichiro Matsuo

**Affiliations:** a School of Pharmaceutical Sciences, University of Shizuoka, 52-1 Yada, Suruga-ku, Shizuoka 422-8526, Japan

## Abstract

The title compound, C_16_H_13_NO_3_, exists in the keto form and the iso­quinoline system is essentially planar (mean deviation = 0.0424 Å). The dihedral angle between the aromatic rings is 4.2 (2)°. In the crystal, mol­ecules are linked *via* weak C—H⋯O hydrogen bonds and C—H⋯π inter­actions, forming a three-dimensional structure.

## Related literature

For the biological properties of the title compound, see: Parkes *et al.* (2003 ▶); Sun *et al.* (2005 ▶); Hang *et al.* (2004 ▶); Billamboz *et al.* (2008 ▶). For related structures, see: Ishikawa & Matsuo (2013 ▶); Lee *et al.* (2008 ▶).
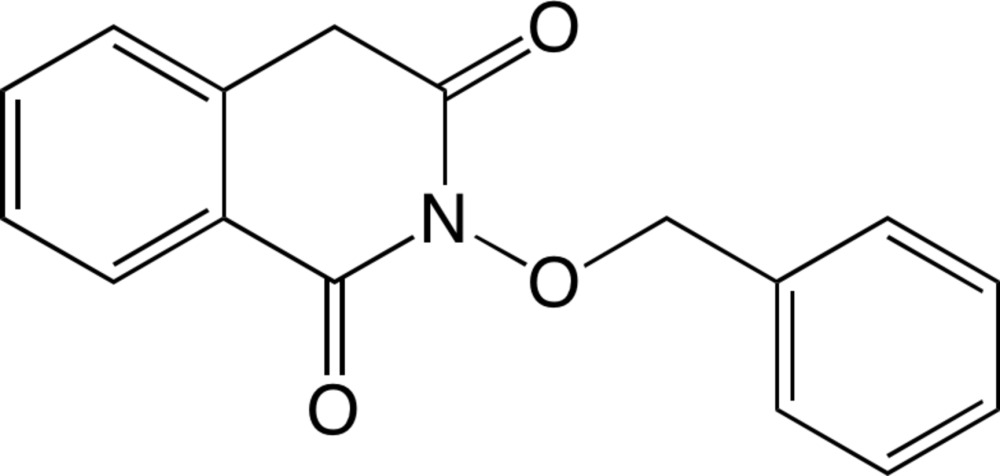


## Experimental

### 

#### Crystal data

C_16_H_13_NO_3_
*M* *_r_* = 267.28
Orthorhombic, 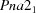

*a* = 7.677 (4) Å
*b* = 12.003 (10) Å
*c* = 13.885 (10) Å
*V* = 1279.4 (15) Å^3^
*Z* = 4
Mo *K*α radiation
μ = 0.10 mm^−1^
*T* = 100 K
0.50 × 0.18 × 0.13 mm

#### Data collection

Rigaku AFC-7R diffractometer
Absorption correction: ψ scan (North *et al.*, 1968 ▶) *T*_min_ = 0.262, *T*_max_ = 0.988
2451 measured reflections
1756 independent reflections
1191 reflections with *F* ^2^ > 2σ(*F* ^2^)
*R*_int_ = 0.081
3 standard reflections every 150 reflections intensity decay: 1.0%

#### Refinement

*R*[*F* ^2^ > 2σ(*F* ^2^)] = 0.069
*wR*(*F* ^2^) = 0.212
*S* = 1.03
1756 reflections
182 parameters
1 restraint
H-atom parameters constrained
Δρ_max_ = 0.36 e Å^−3^
Δρ_min_ = −0.39 e Å^−3^

### 

Data collection: *WinAFC Diffractometer Control Software* (Rigaku, 1999 ▶); cell refinement: *WinAFC Diffractometer Control Software*; data reduction: *WinAFC Diffractometer Control Software*; program(s) used to solve structure: *SHELXS97* (Sheldrick, 2008 ▶); program(s) used to refine structure: *SHELXL97* (Sheldrick, 2008 ▶); molecular graphics: *CrystalStructure* (Rigaku, 2010 ▶); software used to prepare material for publication: *CrystalStructure*.

## Supplementary Material

Crystal structure: contains datablock(s) global, I. DOI: 10.1107/S1600536813019934/zl2556sup1.cif

Structure factors: contains datablock(s) I. DOI: 10.1107/S1600536813019934/zl2556Isup2.hkl

Supplementary material file. DOI: 10.1107/S1600536813019934/zl2556Isup3.cml

Additional supplementary materials:  crystallographic information; 3D view; checkCIF report

## Figures and Tables

**Table 1:** Hydrogen-bond geometry (Å, °) *Cg*2 and *Cg*3 are the centroids of the C4–C8 and C11–C16 rings, respectively.

*D*—H⋯*A*	*D*—H	H⋯*A*	*D*⋯*A*	*D*—H⋯*A*
C6—H25⋯O19^i^	0.95	2.51	3.334 (8)	146
C3—H22*B*⋯*Cg*3^ii^	0.99	2.91	3.556 (7)	124
C7—H26⋯*Cg*2^iii^	0.95	2.88	3.470 (8)	122

Symmetry codes: (i) 
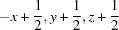
; (ii) 
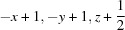
; (iii) 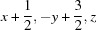.

**Fig. 1.**
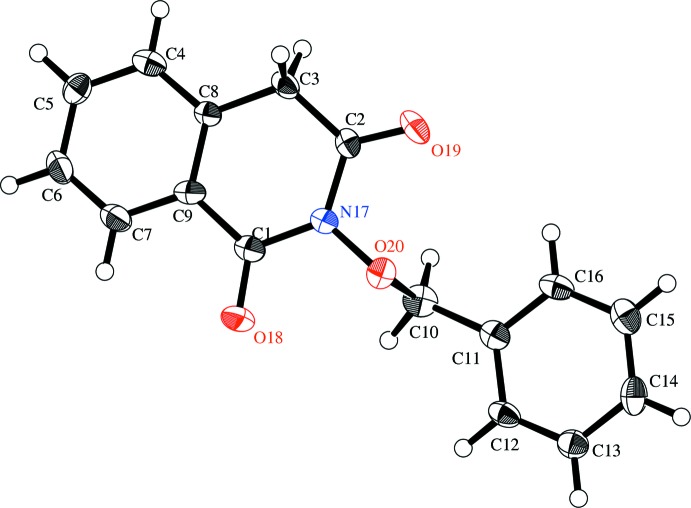
The molecular structure of the title compound, with displacement ellipsoids drawn at the 50% probability level. H atoms are shown as small spheres of arbitrary radius.

**Fig. 2.**
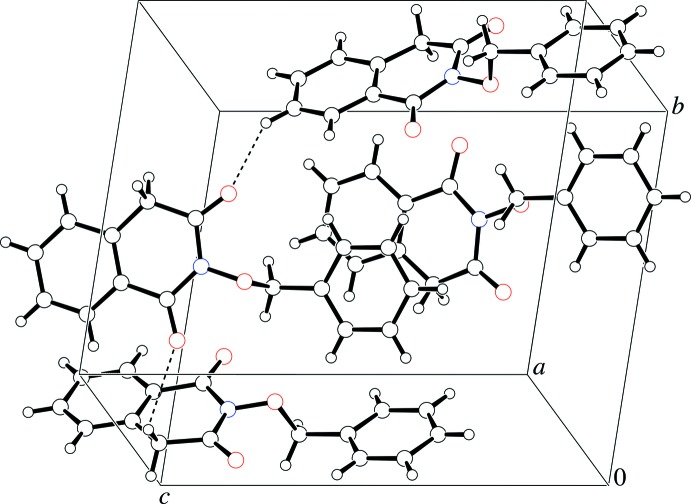
A crystal packing view of the title compound. Intermolecular C–H···O hydrogen bonds are represented as dashed lines.

## supplementary crystallographic information

### Comment

The title compound is a synthetic intermediate of 2-hydroxyisoquinoline-1,3(2*H*,4*H*)-dione known to inhibit metalloenzymes such as influenza endonuclease (Parkes *et al.* 2003), HIV-1 reverse transcriptase RNase H (Hang *et al.* 2004), HIV-1 integrase (Billamboz *et al.* 2008) and *Escherichia coli* ribonuclease III (Sun *et al.* 2005). Here we report the crystal structure of the title compound, which was obtained from the condensation of homophthalic acid with *O*-benzylhydroxylamine using a Dean–Stark apparatus.

The compound, C_16_H_13_NO_3_, crystallizes in the orthorhombic space group *Pna*2_1_. It exists in its keto form [C2—C3—C8 = 117.6 (5)°], as shown in Fig. 1, and the isoquinoline moiety is essentially planar (r.m.s. deviation = 0.0424 Å). The dihedral angle between the least-square planes of the benzene rings is 4.2 (2)°. In the crystal, the molecules are packed through not only intermolecular C—H···O interactions between the isoquinoline methylene H21A^i^ (i: *x* + 1/2, -*y* + 1/2 + 1, *z*) and isoquinoline-1,3(2*H*,4*H*)-dione O18 atoms, and the isoquinoline-1,3(2*H*,4*H*)-dione H25^ii^ (ii: -*x* + 1/2, *y* + 1/2–1, *z* + 1/2–1) and O19 atoms, but also through a C—H···π interaction between the isoquinoline-1,3(2*H*,4*H*)-dione H22B^iii^ (iii: -*x* + 1, -*y* + 1, *z* - 1/2) atom and the benzyl phenyl ring (Fig. 2).

### Experimental

The title compound was synthesized according to the literature (Billamboz *et al.*, 2008). Single crystals suitable for X-ray diffraction were obtained by slow evaporation of an ethyl acetate solution of the compound at room temperature.

### Refinement

The phenyl protons of the isoquinoline ring [C—H = 0.95 Å, *U*_iso_(H) = 1.2*U*_eq_(C)] and the methylene protons of the benzyl group [C—H = 0.99 Å, *U*_iso_(H) = 1.2*U*_eq_(C)] were placed in geometrical positions, and refined using a riding model. The methylene protons of the isoquinoline ring were found in a difference Fourier map, and also refined using a riding model [C—H = 0.99 Å, *U*_iso_(H) = 1.2*U*_eq_(C)].

### Crystal data

**Table:**

C_16_H_13_NO_3_	*F*(000) = 560.00
*M**_r_* = 267.28	*D*_x_ = 1.387 Mg m^−^^3^
Orthorhombic, *P**n**a*2_1_	Mo *K*α radiation, λ = 0.71069 Å
Hall symbol: P 2c -2n	Cell parameters from 24 reflections
*a* = 7.677 (4) Å	θ = 12.7–16.5°
*b* = 12.003 (10) Å	µ = 0.10 mm^−^^1^
*c* = 13.885 (10) Å	*T* = 100 K
*V* = 1279.4 (15) Å^3^	Block, colourless
*Z* = 4	0.50 × 0.18 × 0.13 mm

### Data collection

**Table:**

Rigaku AFC-7R diffractometer	*R*_int_ = 0.081
ω/2θ scans	θ_max_ = 27.5°
Absorption correction: ψ scan (North *et al.*, 1968)	*h* = −5→9
*T*_min_ = 0.262, *T*_max_ = 0.988	*k* = −8→15
2451 measured reflections	*l* = −18→10
1756 independent reflections	3 standard reflections every 150 reflections
1191 reflections with *F*^2^ > 2σ(*F*^2^)	intensity decay: 1.0%

### Refinement

**Table:**

Refinement on *F*^2^	Secondary atom site location: difference Fourier map
*R*[*F*^2^ > 2σ(*F*^2^)] = 0.069	Hydrogen site location: inferred from neighbouring sites
*wR*(*F*^2^) = 0.212	H-atom parameters constrained
*S* = 1.03	*w* = 1/[σ^2^(*F*_o_^2^) + (0.1324*P*)^2^ + 0.3682*P*] where *P* = (*F*_o_^2^ + 2*F*_c_^2^)/3
1756 reflections	(Δ/σ)_max_ < 0.001
182 parameters	Δρ_max_ = 0.36 e Å^−^^3^
1 restraint	Δρ_min_ = −0.39 e Å^−^^3^
Primary atom site location: structure-invariant direct methods	

### Special details

**Table:**

Refinement. Refinement was performed using all reflections. The weighted *R*-factor (*wR*) and goodness of fit (*S*) are based on *F*^2^. *R*-factor (gt) are based on *F*. The threshold expression of *F*^2^ > 2.0 σ(*F*^2^) is used only for calculating *R*-factor (gt).

### Fractional atomic coordinates and isotropic or equivalent isotropic displacement parameters (Å^2^)

**Table:**

	*x*	*y*	*z*	*U*_iso_*/*U*_eq_	
O18	0.3591 (6)	0.8028 (4)	0.3730 (4)	0.0355 (13)	
O19	0.1647 (6)	0.4679 (4)	0.2670 (4)	0.0339 (12)	
O20	0.2975 (6)	0.6634 (4)	0.2295 (3)	0.0260 (11)	
N17	0.2641 (7)	0.6331 (4)	0.3243 (4)	0.0240 (12)	
C1	0.2937 (8)	0.7121 (5)	0.3953 (5)	0.0240 (14)	
C2	0.1810 (7)	0.5289 (5)	0.3351 (5)	0.0207 (14)	
C3	0.1218 (8)	0.5009 (5)	0.4351 (5)	0.0251 (14)	
C4	0.1467 (8)	0.5476 (5)	0.6103 (5)	0.0275 (15)	
C5	0.1916 (9)	0.6178 (5)	0.6834 (5)	0.0282 (15)	
C6	0.2712 (9)	0.7203 (6)	0.6642 (5)	0.0308 (16)	
C7	0.3035 (9)	0.7490 (6)	0.5712 (5)	0.0291 (16)	
C8	0.1770 (7)	0.5759 (5)	0.5142 (5)	0.0199 (13)	
C9	0.2574 (8)	0.6785 (5)	0.4957 (6)	0.0223 (13)	
C10	0.4704 (9)	0.6278 (6)	0.2021 (5)	0.0314 (16)	
C11	0.4750 (8)	0.6293 (5)	0.0941 (5)	0.0242 (15)	
C12	0.5520 (8)	0.7162 (5)	0.0431 (5)	0.0263 (15)	
C13	0.5527 (9)	0.7163 (5)	−0.0557 (5)	0.0278 (15)	
C14	0.4772 (8)	0.6294 (6)	−0.1066 (5)	0.0307 (16)	
C15	0.3991 (8)	0.5420 (6)	−0.0573 (6)	0.0314 (16)	
C16	0.3984 (8)	0.5424 (5)	0.0420 (5)	0.0288 (15)	
H21A	−0.0071	0.4982	0.4349	0.0302*	
H22B	0.1636	0.4249	0.4504	0.0302*	
H23	0.0936	0.4782	0.6251	0.0331*	
H24	0.1686	0.5968	0.7481	0.0338*	
H25	0.3022	0.7690	0.7154	0.0370*	
H26	0.3584	0.8181	0.5575	0.0350*	
H27A	0.4939	0.5518	0.2266	0.0376*	
H28B	0.5591	0.6791	0.2288	0.0376*	
H29	0.6047	0.7760	0.0771	0.0315*	
H30	0.6052	0.7764	−0.0895	0.0334*	
H31	0.4789	0.6296	−0.1750	0.0368*	
H32	0.3466	0.4824	−0.0917	0.0376*	
H33	0.3449	0.4825	0.0756	0.0346*	

### Atomic displacement parameters (Å^2^)

**Table:**

	*U*^11^	*U*^22^	*U*^33^	*U*^12^	*U*^13^	*U*^23^
O18	0.046 (3)	0.020 (3)	0.041 (3)	−0.007 (2)	0.008 (3)	0.001 (3)
O19	0.035 (3)	0.026 (3)	0.040 (3)	−0.005 (2)	−0.004 (3)	−0.012 (3)
O20	0.026 (3)	0.027 (3)	0.025 (3)	0.0063 (18)	−0.002 (2)	0.002 (2)
N17	0.026 (3)	0.021 (3)	0.026 (3)	−0.003 (3)	0.000 (3)	−0.000 (3)
C1	0.016 (3)	0.018 (3)	0.038 (4)	0.004 (3)	0.006 (3)	−0.002 (3)
C2	0.013 (3)	0.022 (3)	0.027 (4)	0.005 (3)	0.000 (3)	−0.006 (3)
C3	0.027 (3)	0.015 (3)	0.033 (4)	0.002 (3)	0.002 (4)	−0.004 (3)
C4	0.023 (4)	0.019 (4)	0.040 (5)	−0.001 (3)	−0.001 (3)	0.002 (3)
C5	0.025 (4)	0.033 (4)	0.027 (4)	0.004 (3)	0.005 (3)	−0.000 (4)
C6	0.031 (4)	0.027 (4)	0.034 (5)	−0.001 (3)	−0.000 (3)	−0.011 (4)
C7	0.028 (4)	0.021 (3)	0.039 (4)	−0.003 (3)	0.003 (3)	−0.003 (4)
C8	0.016 (3)	0.014 (3)	0.030 (4)	0.005 (3)	0.005 (3)	−0.001 (3)
C9	0.015 (3)	0.017 (3)	0.035 (4)	0.003 (3)	0.004 (3)	0.004 (3)
C10	0.024 (4)	0.034 (4)	0.036 (4)	0.001 (3)	−0.006 (3)	0.003 (4)
C11	0.016 (3)	0.021 (4)	0.036 (4)	0.006 (3)	0.002 (3)	−0.002 (3)
C12	0.021 (4)	0.017 (3)	0.041 (4)	0.001 (3)	0.003 (4)	−0.002 (3)
C13	0.028 (4)	0.024 (4)	0.031 (4)	0.001 (3)	−0.001 (3)	0.001 (3)
C14	0.030 (4)	0.035 (4)	0.026 (4)	0.013 (3)	−0.004 (3)	−0.007 (4)
C15	0.024 (4)	0.029 (4)	0.040 (5)	0.008 (3)	−0.001 (4)	−0.008 (4)
C16	0.026 (4)	0.019 (3)	0.042 (5)	−0.002 (3)	−0.000 (4)	0.003 (3)

### Geometric parameters (Å, º)

**Table:**

O18—C1	1.238 (8)	C12—C13	1.372 (10)
O19—C2	1.203 (8)	C13—C14	1.387 (9)
O20—N17	1.389 (7)	C14—C15	1.388 (10)
O20—C10	1.445 (8)	C15—C16	1.379 (10)
N17—C1	1.388 (9)	C3—H21A	0.990
N17—C2	1.411 (8)	C3—H22B	0.990
C1—C9	1.478 (10)	C4—H23	0.950
C2—C3	1.499 (9)	C5—H24	0.950
C3—C8	1.482 (9)	C6—H25	0.950
C4—C5	1.362 (10)	C7—H26	0.950
C4—C8	1.397 (9)	C10—H27A	0.990
C5—C6	1.399 (9)	C10—H28B	0.990
C6—C7	1.360 (10)	C12—H29	0.950
C7—C9	1.392 (10)	C13—H30	0.950
C8—C9	1.401 (8)	C14—H31	0.950
C10—C11	1.500 (9)	C15—H32	0.950
C11—C12	1.392 (9)	C16—H33	0.950
C11—C16	1.400 (9)		
			
O18···O20	2.644 (7)	C12···H22B^xii^	3.2667
O18···C2	3.599 (8)	C12···H22B^viii^	3.0478
O18···C7	2.860 (9)	C12···H23^xii^	3.5261
O18···C10	3.281 (8)	C12···H29^v^	3.4674
O19···O20	2.611 (6)	C13···H21A^xii^	3.4039
O19···C1	3.571 (8)	C13···H22B^xii^	3.0049
O19···C10	3.162 (8)	C13···H22B^viii^	2.7609
O20···C12	3.304 (8)	C13···H30^v^	3.4683
O20···C16	3.081 (8)	C14···H22B^viii^	2.9417
N17···C8	2.806 (9)	C14···H24^xiii^	3.1364
N17···C11	3.582 (9)	C14···H25^xiii^	3.2749
C1···C3	2.912 (9)	C14···H27A^viii^	3.1855
C1···C10	3.172 (10)	C14···H30^v^	3.0805
C2···C9	2.922 (9)	C15···H21A^ii^	3.0498
C2···C10	3.123 (9)	C15···H22B^viii^	3.3817
C4···C7	2.754 (10)	C15···H24^xiii^	3.2965
C5···C9	2.753 (10)	C15···H27A^viii^	3.3091
C6···C8	2.805 (9)	C15···H30^v^	3.1688
C11···C14	2.787 (10)	C16···H21A^ii^	3.3870
C12···C15	2.773 (10)	C16···H26^iii^	3.3432
C13···C16	2.757 (9)	C16···H29^v^	3.1734
O18···N17^i^	3.273 (7)	H21A···O18^v^	2.7383
O18···C1^i^	3.356 (8)	H21A···C13^ix^	3.4039
O18···C2^i^	3.235 (8)	H21A···C15^vi^	3.0498
O18···C3^i^	3.220 (8)	H21A···C16^vi^	3.3870
O18···C8^i^	3.453 (8)	H21A···H24^ii^	3.0922
O18···C9^i^	3.507 (8)	H21A···H26^v^	2.9708
O19···C4^ii^	3.237 (8)	H21A···H29^ix^	3.4020
O19···C5^ii^	3.144 (8)	H21A···H30^ix^	2.7873
O19···C6^iii^	3.334 (8)	H21A···H32^vi^	2.6426
O19···C14^iv^	3.465 (8)	H21A···H33^vi^	3.2553
O20···C10^v^	3.567 (8)	H22B···C11^iv^	3.4786
O20···C12^v^	3.512 (8)	H22B···C12^ix^	3.2667
N17···O18^v^	3.273 (7)	H22B···C12^iv^	3.0478
C1···O18^v^	3.356 (8)	H22B···C13^ix^	3.0049
C2···O18^v^	3.235 (8)	H22B···C13^iv^	2.7609
C2···C14^iv^	3.339 (9)	H22B···C14^iv^	2.9417
C3···O18^v^	3.220 (8)	H22B···C15^iv^	3.3817
C3···C14^iv^	3.501 (9)	H22B···H29^ix^	3.2450
C4···O19^vi^	3.237 (8)	H22B···H29^iv^	3.4746
C5···O19^vi^	3.144 (8)	H22B···H30^ix^	2.7823
C6···O19^vii^	3.334 (8)	H22B···H30^iv^	3.0485
C8···O18^v^	3.453 (8)	H22B···H31^iv^	3.3152
C8···C16^iv^	3.576 (9)	H23···O19^vi^	2.8692
C9···O18^v^	3.507 (8)	H23···C2^vi^	3.6000
C10···O20^i^	3.567 (8)	H23···C11^iv^	3.5800
C12···O20^i^	3.512 (8)	H23···C12^ix^	3.5261
C14···O19^viii^	3.465 (8)	H23···H26^v^	3.1810
C14···C2^viii^	3.339 (9)	H23···H27A^iv^	3.4851
C14···C3^viii^	3.501 (9)	H23···H28B^iv^	3.5703
C16···C8^viii^	3.576 (9)	H23···H29^ix^	2.9408
O18···H26	2.5693	H23···H33^vi^	3.4679
O18···H28B	2.9272	H24···O19^vi^	2.6864
O19···H21A	2.7032	H24···C2^vi^	3.3076
O19···H22B	2.5992	H24···C14^xi^	3.1364
O19···H27A	2.7774	H24···C15^xi^	3.2965
O19···H33	3.0006	H24···H21A^vi^	3.0922
O20···H29	3.4447	H24···H25^v^	3.2725
O20···H33	3.0680	H24···H27A^iv^	3.1607
N17···H21A	3.0517	H24···H30^x^	2.7632
N17···H22B	3.1476	H24···H31^xi^	2.6402
N17···H27A	2.4292	H24···H32^xi^	2.9496
N17···H28B	2.6821	H25···O19^vii^	2.5062
C1···H21A	3.4970	H25···C2^vii^	3.5380
C1···H26	2.6337	H25···C5^i^	3.3133
C1···H27A	3.3989	H25···C14^xi^	3.2749
C1···H28B	3.1074	H25···H24^i^	3.2725
C2···H27A	2.8484	H25···H30^xi^	3.5721
C3···H23	2.6606	H25···H30^x^	3.1509
C4···H21A	2.7715	H25···H31^xi^	2.6373
C4···H22B	2.6683	H25···H31^x^	3.1564
C4···H25	3.2573	H25···H33^vii^	3.4076
C5···H26	3.2367	H26···O19^vii^	3.4239
C6···H23	3.2552	H26···C3^i^	3.4205
C7···H24	3.2315	H26···C4^i^	2.8340
C8···H24	3.2589	H26···C5^i^	3.1922
C8···H26	3.2792	H26···C6^i^	3.5289
C9···H21A	3.0855	H26···C7^i^	3.5159
C9···H22B	3.1913	H26···C8^i^	2.8223
C9···H23	3.2542	H26···C9^i^	3.1811
C9···H25	3.2556	H26···C16^vii^	3.3432
C10···H29	2.6909	H26···H21A^i^	2.9708
C10···H33	2.6560	H26···H23^i^	3.1810
C11···H30	3.2581	H26···H32^vii^	3.2649
C11···H32	3.2770	H26···H33^vii^	2.5284
C12···H27A	3.2528	H27A···C4^viii^	3.4128
C12···H28B	2.6163	H27A···C5^viii^	3.2149
C12···H31	3.2506	H27A···C14^iv^	3.1855
C12···H33	3.2552	H27A···C15^iv^	3.3091
C13···H32	3.2611	H27A···H23^viii^	3.4851
C14···H29	3.2500	H27A···H24^viii^	3.1607
C14···H33	3.2461	H27A···H31^iv^	2.5797
C15···H30	3.2584	H27A···H32^iv^	2.8344
C16···H27A	2.6682	H28B···O18^i^	3.0594
C16···H28B	3.3079	H28B···O20^i^	2.6303
C16···H29	3.2576	H28B···N17^i^	3.0521
C16···H31	3.2488	H28B···C1^i^	3.2085
H21A···H23	2.7623	H28B···H23^viii^	3.5703
H22B···H23	2.5650	H28B···H32^iv^	3.2401
H23···H24	2.2976	H29···O20^i^	2.6822
H24···H25	2.3513	H29···C10^i^	3.4963
H25···H26	2.3102	H29···C11^i^	3.0703
H27A···H29	3.5035	H29···C12^i^	3.4674
H27A···H33	2.5284	H29···C16^i^	3.1734
H28B···H29	2.4309	H29···H21A^xii^	3.4020
H28B···H33	3.5769	H29···H22B^xii^	3.2450
H29···H30	2.3133	H29···H22B^viii^	3.4746
H30···H31	2.3353	H29···H23^xii^	2.9408
H31···H32	2.3427	H29···H33^i^	3.4347
H32···H33	2.3236	H30···C3^xii^	3.2264
O18···H21A^i^	2.7383	H30···C5^xiv^	3.4640
O18···H28B^v^	3.0594	H30···C13^i^	3.4683
O18···H32^vii^	2.7174	H30···C14^i^	3.0805
O19···H23^ii^	2.8692	H30···C15^i^	3.1688
O19···H24^ii^	2.6864	H30···H21A^xii^	2.7873
O19···H25^iii^	2.5062	H30···H22B^xii^	2.7823
O19···H26^iii^	3.4239	H30···H22B^viii^	3.0485
O19···H31^iv^	3.0836	H30···H24^xiv^	2.7632
O20···H28B^v^	2.6303	H30···H25^xiii^	3.5721
O20···H29^v^	2.6822	H30···H25^xiv^	3.1509
N17···H28B^v^	3.0521	H30···H31^i^	3.3032
N17···H32^iv^	3.4949	H30···H32^i^	3.4373
C1···H28B^v^	3.2085	H31···O19^viii^	3.0836
C1···H32^vii^	3.4231	H31···C2^viii^	3.2337
C2···H23^ii^	3.6000	H31···C5^xiii^	2.9585
C2···H24^ii^	3.3076	H31···C6^xiii^	2.9513
C2···H25^iii^	3.5380	H31···C10^viii^	3.5514
C2···H31^iv^	3.2337	H31···H22B^viii^	3.3152
C3···H26^v^	3.4205	H31···H24^xiii^	2.6402
C3···H30^ix^	3.2264	H31···H25^xiii^	2.6373
C4···H26^v^	2.8340	H31···H25^xiv^	3.1564
C4···H27A^iv^	3.4128	H31···H27A^viii^	2.5797
C5···H25^v^	3.3133	H31···H30^v^	3.3032
C5···H26^v^	3.1922	H32···O18^iii^	2.7174
C5···H27A^iv^	3.2149	H32···N17^viii^	3.4949
C5···H30^x^	3.4640	H32···C1^iii^	3.4231
C5···H31^xi^	2.9585	H32···C10^viii^	3.4525
C6···H26^v^	3.5289	H32···H21A^ii^	2.6426
C6···H31^xi^	2.9513	H32···H24^xiii^	2.9496
C6···H33^vii^	3.4955	H32···H26^iii^	3.2649
C7···H26^v^	3.5159	H32···H27A^viii^	2.8344
C7···H33^vii^	3.0267	H32···H28B^viii^	3.2401
C8···H26^v^	2.8223	H32···H30^v^	3.4373
C9···H26^v^	3.1811	H33···C6^iii^	3.4955
C10···H29^v^	3.4963	H33···C7^iii^	3.0267
C10···H31^iv^	3.5514	H33···H21A^ii^	3.2553
C10···H32^iv^	3.4525	H33···H23^ii^	3.4679
C11···H22B^viii^	3.4786	H33···H25^iii^	3.4076
C11···H23^viii^	3.5800	H33···H26^iii^	2.5284
C11···H29^v^	3.0703	H33···H29^v^	3.4347
			
N17—O20—C10	109.9 (5)	C11—C16—C15	121.2 (6)
O20—N17—C1	117.6 (5)	C2—C3—H21A	107.914
O20—N17—C2	114.7 (5)	C2—C3—H22B	107.913
C1—N17—C2	127.1 (6)	C8—C3—H21A	107.922
O18—C1—N17	119.3 (6)	C8—C3—H22B	107.920
O18—C1—C9	123.6 (6)	H21A—C3—H22B	107.196
N17—C1—C9	116.9 (6)	C5—C4—H23	119.363
O19—C2—N17	120.1 (6)	C8—C4—H23	119.352
O19—C2—C3	124.0 (6)	C4—C5—H24	119.596
N17—C2—C3	115.8 (5)	C6—C5—H24	119.593
C2—C3—C8	117.6 (5)	C5—C6—H25	120.560
C5—C4—C8	121.3 (6)	C7—C6—H25	120.552
C4—C5—C6	120.8 (7)	C6—C7—H26	119.495
C5—C6—C7	118.9 (7)	C9—C7—H26	119.505
C6—C7—C9	121.0 (7)	O20—C10—H27A	110.462
C3—C8—C4	120.8 (6)	O20—C10—H28B	110.463
C3—C8—C9	121.6 (6)	C11—C10—H27A	110.467
C4—C8—C9	117.5 (6)	C11—C10—H28B	110.470
C1—C9—C7	119.8 (6)	H27A—C10—H28B	108.651
C1—C9—C8	119.7 (6)	C11—C12—H29	119.632
C7—C9—C8	120.5 (7)	C13—C12—H29	119.618
O20—C10—C11	106.3 (5)	C12—C13—H30	119.774
C10—C11—C12	121.8 (6)	C14—C13—H30	119.782
C10—C11—C16	119.9 (6)	C13—C14—H31	120.076
C12—C11—C16	118.3 (6)	C15—C14—H31	120.079
C11—C12—C13	120.7 (6)	C14—C15—H32	120.249
C12—C13—C14	120.4 (6)	C16—C15—H32	120.252
C13—C14—C15	119.8 (7)	C11—C16—H33	119.416
C14—C15—C16	119.5 (7)	C15—C16—H33	119.405
			
N17—O20—C10—C11	162.1 (4)	C5—C6—C7—H26	−179.5
N17—O20—C10—H27A	42.2	H25—C6—C7—C9	−179.5
N17—O20—C10—H28B	−78.0	H25—C6—C7—H26	0.5
C10—O20—N17—C1	95.6 (5)	C6—C7—C9—C1	179.9 (6)
C10—O20—N17—C2	−92.6 (5)	C6—C7—C9—C8	−0.3 (9)
O20—N17—C1—O18	−4.9 (8)	H26—C7—C9—C1	−0.1
O20—N17—C1—C9	179.9 (4)	H26—C7—C9—C8	179.7
O20—N17—C2—O19	9.4 (7)	C3—C8—C9—C1	−2.0 (8)
O20—N17—C2—C3	−172.1 (4)	C3—C8—C9—C7	178.1 (5)
C1—N17—C2—O19	−179.7 (5)	C4—C8—C9—C1	179.5 (5)
C1—N17—C2—C3	−1.2 (8)	C4—C8—C9—C7	−0.3 (8)
C2—N17—C1—O18	−175.6 (5)	O20—C10—C11—C12	100.9 (6)
C2—N17—C1—C9	9.2 (9)	O20—C10—C11—C16	−78.3 (6)
O18—C1—C9—C7	−2.5 (9)	H27A—C10—C11—C12	−139.2
O18—C1—C9—C8	177.6 (5)	H27A—C10—C11—C16	41.6
N17—C1—C9—C7	172.4 (5)	H28B—C10—C11—C12	−19.0
N17—C1—C9—C8	−7.4 (8)	H28B—C10—C11—C16	161.9
O19—C2—C3—C8	170.0 (5)	C10—C11—C12—C13	−179.2 (5)
O19—C2—C3—H21A	−67.8	C10—C11—C12—H29	0.8
O19—C2—C3—H22B	47.8	C10—C11—C16—C15	179.4 (5)
N17—C2—C3—C8	−8.4 (7)	C10—C11—C16—H33	−0.6
N17—C2—C3—H21A	113.8	C12—C11—C16—C15	0.2 (9)
N17—C2—C3—H22B	−130.7	C12—C11—C16—H33	−179.8
C2—C3—C8—C4	−171.6 (5)	C16—C11—C12—C13	−0.0 (9)
C2—C3—C8—C9	10.1 (8)	C16—C11—C12—H29	180.0
H21A—C3—C8—C4	66.2	C11—C12—C13—C14	−0.3 (9)
H21A—C3—C8—C9	−112.2	C11—C12—C13—H30	179.7
H22B—C3—C8—C4	−49.3	H29—C12—C13—C14	179.7
H22B—C3—C8—C9	132.3	H29—C12—C13—H30	−0.3
C5—C4—C8—C3	−177.7 (5)	C12—C13—C14—C15	0.5 (9)
C5—C4—C8—C9	0.7 (9)	C12—C13—C14—H31	−179.5
C8—C4—C5—C6	−0.5 (9)	H30—C13—C14—C15	−179.5
C8—C4—C5—H24	179.5	H30—C13—C14—H31	0.5
H23—C4—C5—C6	179.5	C13—C14—C15—C16	−0.3 (9)
H23—C4—C5—H24	−0.5	C13—C14—C15—H32	179.7
H23—C4—C8—C3	2.3	H31—C14—C15—C16	179.6
H23—C4—C8—C9	−179.3	H31—C14—C15—H32	−0.4
C4—C5—C6—C7	−0.1 (9)	C14—C15—C16—C11	−0.0 (9)
C4—C5—C6—H25	179.9	C14—C15—C16—H33	180.0
H24—C5—C6—C7	179.9	H32—C15—C16—C11	180.0
H24—C5—C6—H25	−0.1	H32—C15—C16—H33	−0.0
C5—C6—C7—C9	0.5 (10)		

Symmetry codes: (i) *x*+1/2, −*y*+3/2, *z*; (ii) −*x*, −*y*+1, *z*−1/2; (iii) −*x*+1/2, *y*−1/2, *z*−1/2; (iv) −*x*+1, −*y*+1, *z*+1/2; (v) *x*−1/2, −*y*+3/2, *z*; (vi) −*x*, −*y*+1, *z*+1/2; (vii) −*x*+1/2, *y*+1/2, *z*+1/2; (viii) −*x*+1, −*y*+1, *z*−1/2; (ix) −*x*+1/2, *y*−1/2, *z*+1/2; (x) *x*−1/2, −*y*+3/2, *z*+1; (xi) *x*, *y*, *z*+1; (xii) −*x*+1/2, *y*+1/2, *z*−1/2; (xiii) *x*, *y*, *z*−1; (xiv) *x*+1/2, −*y*+3/2, *z*−1.

### Hydrogen-bond geometry (Å, º)

Cg2 and Cg3 are the centroids of the C4–C8 and C11–C16 rings, respectively.

**Table:**

*D*—H···*A*	*D*—H	H···*A*	*D*···*A*	*D*—H···*A*
C3^i^—H21*A*^i^···O18	0.99	2.74	3.220 (8)	110
C6—H25···O19^vii^	0.95	2.51	3.334 (8)	146
C3—H22*B*···*Cg*3^iv^	0.99	2.91	3.556 (7)	124
C7—H26···*Cg*2^i^	0.95	2.88	3.470 (8)	122

Symmetry codes: (i) *x*+1/2, −*y*+3/2, *z*; (iv) −*x*+1, −*y*+1, *z*+1/2; (vii) −*x*+1/2, *y*+1/2, *z*+1/2.
